# Harsh parenting and suicide ideation among Chinese adolescents: the roles of self-esteem and school social support

**DOI:** 10.1186/s12888-023-05108-w

**Published:** 2023-08-21

**Authors:** Jingfei Zhao, Yulong Wang

**Affiliations:** School of Educational Science, Cognition and Hunan Behavior Key Laboratory of Hunan Province, Research Center for Mental Health Education of Hunan Province, Hunan Normal University, Changsha, Hunan 410081 China

**Keywords:** Harsh parenting, Suicide ideation, Self-esteem, School social support

## Abstract

**Background:**

While negative parenting style has considered as a risk factor for suicide ideation, little attention has been given to the mechanisms between harsh parenting and suicide ideation in the context of Chinese culture. This study explored the the potential mediating roles of self-esteem and the potential moderating roles of school social support in the relationship between harsh parenting and suicide ideation among Chinese adolescents.

**Methods:**

We conducted a cross-sectional study among 4189 Chinese adolescents who completed measures of harsh parenting, school social support, self-esteem, and suicide ideation. The moderated mediation model was used to test the roles of self-esteem and school social support on the association between harsh parenting and adolescent suicide ideation.

**Results:**

(1) There were significant positive correlations between harsh parenting and adolescents suicide ideation. (2) Self-esteem mediates the relationship between harsh parenting and suicide ideation in adolescents. (3) School social support moderates the indirect effect of harsh parenting on suicidal ideation.

**Conclusions:**

Beyond the direct impact of harsh parenting, harsh parenting also indirectly contributes to adolescent suicide ideation via the mediator of adolescents’ self-esteem. School social support moderates the relationship between harsh parenting and self-esteem as well as the relationship between self-esteem and suicide ideation. The findings suggest potential pathways for suicide prevention and intervention strategies and highlighted that buffering effect of school social support is limited as risk increasing.

## Introduction

As a serious global public health issue, suicide is the fourth leading cause of mortality in 15–19-year-olds [1]. Suicide ideation is defined as thoughts about engaging in suicidal behavior, which confers risk for suicide attempts and completed suicide [2]. Suicide ideation rates increase during early adolescence and reaching a peak during mid-adolescence [3]. Besides contributing to mortality outcome, suicide ideation in adolescence can produce negative consequences that last far into adulthood. Adolescents who reported suicidal ideation have a higher probability of psychiatric disorders, problem behaviors and compromised functioning [4, 5]. It is crucial, therefore, to better understand the factors that induce adolescents to consider suicide.

Harsh parenting typically involves parental discipline behaviors instance of yelling, spanking, slapping, shoving, or hitting the child with an object [6], which is characterized by a low acceptance and high strictness and driven more by the parent’s emotional reactions rather than the child’s interests [7]. It reaches lower degrees of severity than child maltreatment, with verbally-abusive parenting (e.g., shouting, threatening, or yelling), and physically-abusive parenting (e.g., spanking) being the most universal forms [8]. There is broad consensus on the harmful impacts of severe forms of child maltreatment, but there is an ongoing debate on the effects of the less severe forms of harsh parenting. Traditional Chinese cultures regard harsh parenting behaviors as a sign of parental involvement and concern, harsh parenting is commonly accepted and ubiquitous in China [9]. Wang and Liu (2014) found that about 48–80% of mainland Chinese parents used psychological aggression or corporal punishment in the previous year [9]. According to the interpersonal psychological theory of suicide, suicidal desire occurs when an individual experiences thwarted belongingness and perceived burdensomeness [10]. Adolescents who are subjected to harsh parenting may feel insecure in the family, develop a frustrated sense of belonging to the family, and perceive themselves as a burden or disappointment to others, all of which can trigger suicidal ideation. There is compelling evidence that children exposed to harsh parenting are at risk for a myriad of adverse developmental outcomes such as anxiety, depression, negative self-evaluations, and internalizing problem behaviors [11–14]. These variables are in turn associated with suicide ideation [15]. Thus, harsh parenting may increase the odds of suicide ideation.

Considering the high prevalence and potential hazards of harsh parenting, it is necessary to further investigate the effect of harsh parenting in the context of Chinese culture. Despite harsh parenting may lead to suicide ideation directly, not all children who face such stressors will develop suicide ideation. Suicidal ideation, according to the stress-vulnerability model, is determined not merely by the stressor but also by a multidimensional process that evolves through ongoing transactions of various variables such as personal traits and social support [16, 17]. However, little research has explored the potential pathways in which harsh parenting could be related to adolescent suicide ideation, especially in the Chinese cultural context. The current study intends to analyse the association between harsh parenting and adolescent suicide ideation, with special attention to the roles of self-esteem and school support in this association, in order to provide effective suggestions for the prevention and intervention of adolescent suicide.

### The mediating role of self-esteem

Self-esteem refers to an individual’s overall evaluation of self, closely correlated with perceptions of others’ evaluations of oneself [18], which reflects a person’s relational value, which will increase when one being liked and accepted and decrease when one being rejected and excluded [19, 20]. As such, self-esteem is a construct saturated with both belongingness and burdensomeness and has been shown to be affected by parenting behavior [21, 22]. Negative feedback from significant others provides information that children many internalize, which lays the foundation for self-perceived incompetence and low self-worth [23]. Previous research has suggested that harsh parenting may be detrimental to the self-esteem. For instance, one study by Tang et al. (2018) revealed that when adolescents frequently suffered from harsh parenting, they are more likely to form nagtive self-congnition [24]. Cole et al. (2016) demonstrated that harsh parenting predict the construction of depressive self-schemas in children and adolescents [11].

As an essential part of the individual’s self-concept, self-esteem is a protective factor linked to psychological functioning, which directly related to mental health during adolescence [25] and significantly predicted suicidal ideation [26]. For example, a cross-sectional study on teenagers in Vietnam discovered the connection between low self-esteem and suicidal ideation [27]. Similarly, a longitudinal study by Reid-Russell et al. (2021) suggested that implicit self-esteem is a potential mechanism linking childhood abuse to depression and suicidal ideation [28]. Based on the literature, it seems reasonable to infer that harsh parenting may precede adolescents low self-esteem, ultimately leading to suicide ideation.

### The moderating role of school social support

Social support is a protective factor in students’ lives. The perception of social support could attenuate the adverse psychological effects of stressful events by strengthening internal resources for coping with adversity [29]. School-aged youths spend more time in school than in any other place except at home. School is an essential developmental context for adolescents where teachers and peers foster an environment that is responsive to basic and developmental needs (e.g., interpersonal support) of adolescents [30, 31]. School social support provides a potential opportunity for adolescents to escape negative parenting and fosters a sense of belonging and connectedness that may compensate for a lack of parental support.

Years of research have shown that the social support adolescents perceive from teachers and peers has a profound influence on mental health; it has been correlated negatively with depression, suicide ideation, and suicide attempts; and related positively with self-esteem, social skills and adaptive skills [32–35]. For instance, the research conducted by Tang et al. (2018) found that peer acceptance lessened the deleterious effects of harsh parenting on adolescent depression by buffering the effect of negative self-cognition on depression [24]. Likewise, a recent study by Zhang et al. (2021) suggested that school belonging could moderate the direct relationship between emotional abuse and adolescent suicide ideation [36]. Therefore, it is conceivable that school social support might compromise the negative impact of harsh parenting on adolescent self-esteem, also, school social support may abate the association between poor self-esteem and suicide ideation.

### The present study

Aimed to further explore the relationship between harsh parenting and suicide ideation, the current study tested the mediation effects of self-esteem and the moderation effects of school social support involved in this relationship among adolescents in Chinese context. Based on the previous studies, three hypotheses are formulated: (1) harsh parenting positively associated with adolescent suicide ideation;(2) self-esteem mediate the association between harsh parenting and suicide ideation; (3) the indirect effect of harsh parenting on suicide ideation via self-esteem is moderated by school social support.

## Method

### Participants

Convenience sampling method was adopted to sample Grade 7–12 students from 6 public schools in Changsha and Huaihua, Hunan province. A total of 5104 questionnaires were distributed, and 4189 valid responses were received with a response rate of 82%. A total of 4189 students participated in this study, 2209 males (52.7%), 1976 females (47.2%), and 4 were missing gender information. Participants in the sample ranged in age from 10 to 20 years, the mean age was 13.94 years (SD = 1.73). Nearly 85% of fathers and 87% of the mothers had an educational level of high school or below. In response to one question about their subjective family economic situations, 2% of participants perceived their families as very bad, 6% as bad, 69% as average, 21% as good, and 2% as very good. Data were collected in Hunan during the fall of 2020. Informed consents were obtained from students and their caregivers before the survey. The privacy and anonymity of their answers were guaranteed.

## Measures

### Suicide ideation

Chinese revision of Positive and Negative Suicide Ideation (PANSI) Inventory was employed to assess the frequency of suicide ideation. The scale was developed by Osman et al. (1998) [37] and revised by Wang et al. (2011) [38]. The PANSI is a 14-item self-report instrument that composed of two sub-scale: positive ideation (PANSI-PI; 6 items) and negative suicide ideation (PANSI-NSI; 8 items). Each item is rated on a 5-point Likert scale that ranges from 1 (none of the time) to 5 (most of the time). The total score is obtained by summing all items after reverse scoring the PANSI-PI items. Higher scores represent more suicide ideation. Cronbach’s alpha in this study was 0.91.

### Harsh parenting

The Harsh Discipline Scale revised by Wang (2017) [39] was employed to examine harsh parenting. Participants completed a 4-item scale for each of their parents (e.g.,When you did something wrong, how often did your mom (dad) lose her temper and yell at you?; When you did something wrong, how often did your mom (dad) tell you to get out or lock you out of the house?). Each item was rated on a five-point Likert scale (1 = never, 5 = always). Higher scores indicate harsher parenting. Cronbach’s alpha in this study was 0.87.

### Self-esteem

Rosenberg Self-Esteem Scale (RSES) was used to measure adolescents’ global feelings of self-worth [18]. The scale consists of 10 items, and each item was rated on a five-point Likert scale (1 = strongly disagree, 5 = strongly agree). Higher scores indicate higher self-esteem. Cronbach’s alpha in this study was 0.89.

### School social support

The Perceived School Climate Scale revised by Jia et al. [40] was used to measure the frequency of perceived teacher support (7 items) and peer support (13 items). All items have a 4-point response scale (1 = never, 4 = always). Higher values indicate higher levels of school social support. Confirmatory confirmatory factor analysis results demonstrated that the model fit the data satisfactorily: X^2^/df = 449.15/124, CFI = 0.99, RMSEA = 0.025, demonstrated validity of the tool. Cronbach’s alpha in this study was 0.90.

### Covariates

Previous studies have indicated that adolescent’s gender, age, and family socioeconomic status (SES; e.g., parental education attainment ) would likely affect the study variables [41], thus they were controlled as covariates in this study. Maternal and paternal educational attainment were coded from 1 (equal to or below primary school) to 5 (graduate education or above). Perceived economic situation was assessed by asking the participant to indicate their family’s economic situation from 1(extremely bad) to 5 (very good). Parental educational attainment and perceived economic situation were used to index adolescent’s SES, and higher scores reflected higher SES.

### Data analysis

In Step 1, the means, standard deviations, and correlations among the variables were computed in SPSS 26.

In step 2, the mediation model (Model 4) was tested using the PROCESS macro [42], to explore whether self-esteem mediates the relationship between harsh parenting and suicide ideation. Age, gender, and SES were included as control variables. All study variables were standardized before model analyses.

In step 3, the moderated mediation model (Model 59) was tested using the PROCESS macro [42], to test whether the indirect effect of harsh parenting on suicide ideation via self-esteem is moderated by school social support. Age, gender, and SES were included as control variables. All study variables were standardized before model analyses.

## Results

### Common method bias

Harman’s single-factor test [43] was used to test common method bias; the results showed nine factors with characteristic roots more significant than 1. The first principal factor explained 26.38% of the variance (< 40%), which meant that our study had no serious standard method bias.

### Descriptive statistics and correlations

The means, standard deviations (SD), and correlation coefficients for all variables considered in this study are indicated in Table 1. Suicide ideation was positively associated with harsh parenting (*r* = 0.38, *p* < 0.001), negatively associated with self-esteem (*r* = -0.72, *p* < 0.001) and school social support (*r* = -0.49, *p* < 0.001). Significant negative correlations were found between harsh parenting and self-esteem (*r* = -0.30, *p* < 0.001).

**Table 1:** Means, Standard Deviations and Correlations for the Variables

	1	2	3	4	5	6	7
1 Age	1						
2 Gender^a^	-0.03	1					
3 SES	-0.10^***^	-0.05^**^	1				
4 Harsh parenting	-0.01	-0.06^***^	0.00	1			
5 Self-esteem	-0.09^***^	-0.05^**^	0.13^***^	-0.30^***^	1		
6 School social support	-0.18^***^	0.06^***^	0.05^**^	-0.32^***^	0.41^***^	1	
7 Suicide ideation	0.10^***^	0.09^***^	-0.08^***^	0.38^***^	-0.72^***^	-0.49^***^	1
M	13.94	-	8.10	13.12	34.97	60.20	27.46
SD	1.73	-	1.85	5.23	8.27	9.39	9.11

Note. N = 4189, ^a^ Male = 0, Female = 1. *p < 0.05. **p < 0.01. ***p < 0.001

Age was significantly associated with self-esteem, school social support and suicide ideation. Younger adolescents report higher self-esteem, higher school social support and lower level of suicide ideation. There were significant gender differences in harsh parenting, self-esteem, school social support and suicide ideation. The male reported more hash parenting (M_1_ = 13.41), higher self-esteem (M_2_ = 35.29), less school social support (M_3_ = 59.65) and lower level of suicide ideation (M_4_ = 26.68) than the female (M_1_ = 12.80, M_2_ = 34.50, M_3_ = 60.83, M_4_ = 28.34).

### The mediating role of self-esteem

The results of the self-esteem mediation test are displayed in Table 2. Consistent with the first hypothesis, harsh parenting had a significant direct path to suicide ideation (b = 0.39, *SE* = 0.01, *p* < 0.001). When self-esteem was included in the model, harsh parenting was still significantly associated with suicide ideation ( *b* = 0.19, *SE* = 0.01, *p <* 0.001); harsh parenting negatively correlated with self-esteem *(b* = -0.30, S*E* = 0.01, *p <* 0.001); self-esteem negatively correlated with suicide ideation (*b* = -0.65, *SE* = 0.01, *p <* 0.001). The indirect effect between harsh parenting and suicide ideation via self-esteem was significant (α * b = 0.20, *95% CI* [0.17, 0.22]) and the indirect effect’s proportion of the total effect was 51.28%, which supported the second hypothesis, self-esteem partially mediated the relation between harsh parenting and suicide ideation.

**Table 2:** The mediating effect of self-esteem in the relation between harsh parenting and suicide ideation

	Model 1 (SI)		Model 2 (SE)		Model 3 (SI)	
	*b*	*t*	*b*	*t*	*b*	*t*
Age	0.06	7.31^***^	-0.05	-5.57^***^	0.03	4.78^***^
Gender	0.23	8.04^***^	-0.14	-4.67^***^	0.14	6.62^***^
SES	-0.03	-4.89^*^	0.06	8.44**	-0.01	1.15
Harsh parenting	0.39	27.27^***^	-0.30	-20.55^***^	0.19	17.29^***^
Self-esteem					-0.65	-60.01^***^
*R* ^*2*^	0.17		0.12		0.56	
*F*	217.91^***^		138.26^***^		1044.65^***^	

Note. N = 4189. b = non-standardized regression coefficient. *p < 0.05. **p < 0.01. ***p < 0.001

### The moderating role of school social support

As displayed in Table 3, the first model provided a significant harsh parenting and school social support interaction effect on adolescents’ self-esteem (*b* = − 0.04, *p* < 0.01), suggesting that school social support would buffer the association between harsh parenting to self-esteem. The second model provided a significant self-esteem and school social support interaction effect on adolescent suicide ideation (*b* = 0.06, *p* < 0.001), suggesting that the indirect effect from self-esteem to suicide ideation is moderated by school social support. The analysis supported the third hypothesis that school social support moderate the indirect association between harsh parenting and suicidal ideation via self-esteem.

**Table 3:** The moderating effect of school social support

	Model 1 (self-esteem)		Model 2 (suicide ideation)	
	*b*	*t*	*b*	*t*
Gender	-0.17	-6.00^***^	0.16	8.11^***^
Age	-0.01	-1.49	0.01	2.32^*^
SES	0.05	7.98^***^	0.01	1.12
Harsh parenting	-0.20	-13.39^***^	0.14	12.03^***^
School social support	0.35	23.66^***^	-0.19	-17.01^***^
Harsh parenting × School social support	-0.04	-3.26^**^	-0.01	-1.57
Self-esteem			-0.59	-52.52^***^
Self-esteem × School social support			0.06	6.55^***^
*R* ^*2*^	0.22		0.59	
*F*	199.17^***^		753.73^***^	

Note: N = 4189. Analyses conducted using PROCESS model 59; b = non-standardized regression coefficient. *p < 0.05. **p < 0.01. ***p < 0.001

**Fig. 1:**
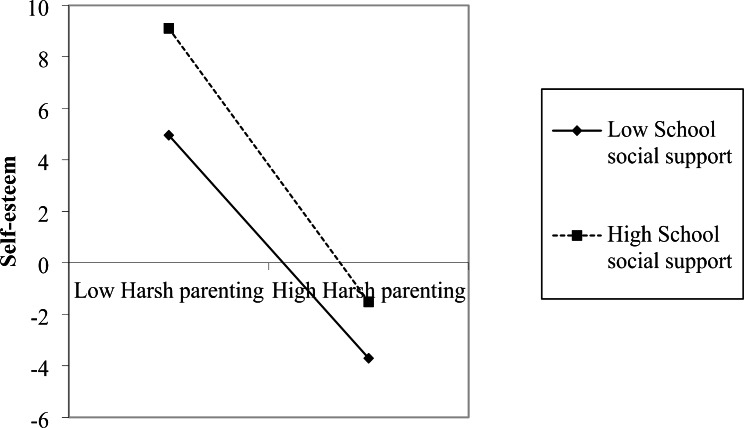
The moderating effect of school social support on the relationship between harsh parenting and self-esteem

**Fig. 2:**
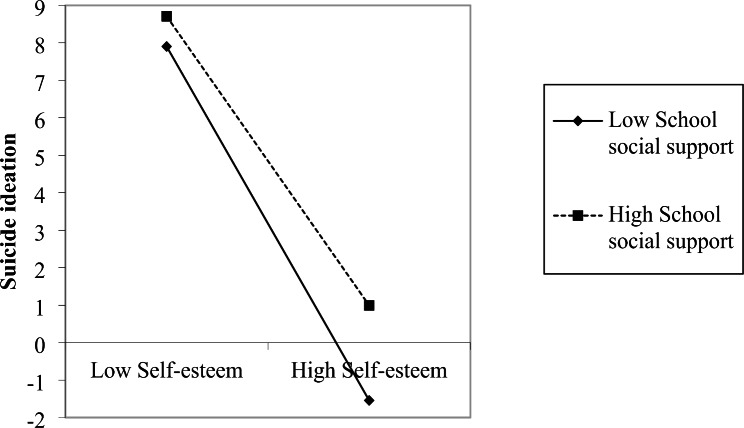
The moderating effect of school social support on the relationship between self-esteem and suicide ideation

Simple slope tests demonstrated that harsh parenting significantly negatively correlated with self-esteem (*bsimple* = − 0.17, *t* = − 10.32, *p* < 0.001) at low level of school social support (*M* − 1*SD*); this indirect effect was stronger(*bsimple* = − 0.24, *t* = − 11.08, *p* < 0.001)at high level of school social support (*M* + 1*SD*) (See Fig. 1). For perceived low school social support individuals, higher self-esteem was associated with lower suicide ideation (*bsimple* = − 0.65, *t* = − 45.57, *p* < 0.001); for perceived high school social support individuals, the effect of self-esteem on suicide ideation was weaker (*bsimple* = − 0.52, *t* = − 34.70, *p* < 0.001) (See Fig. 2).

## Discussion

The current study shed light on the mechanisms underlying the relationship between harsh parenting and adolescent suicide ideation. The results demonstrated that in addition to the direct effect, the contribution of harsh parenting to adolescent suicide ideation could be explicated partially by impaired self-esteem. Moreover, the moderating roles of perceived school social support were manifested in the indirect relations between harsh parenting and adolescents’ suicide ideation via self-esteem.

The present study showed that harsh parenting is a risk factor for adolescent suicide ideation, strengthened existing research linking negative parenting styles to suicide ideation [36, 44, 45]. Chinese parents endorse higher levels of physical punishment and harsh discipline and express their warmth more subtly and implicitly [46]. Some features of parental strictness that Western children interpret as oppression and hostility may be perceived as concern and care in Chinese culture [47]. Chinese children, however, are not immune to the negative effects of harsh parenting. Our results backed up the interpersonal psychological theory of suicide [10], which states that parenting processes are the crucial type of interpersonal interaction, and harsh parenting may impair teenagers’ feelings of belonging and foster interpersonal insecurity. Adolescents who perceived low parental warmth and high controlling had more suicide ideation than those with better-functioning families [49, 50]. Contemporary Chinese parenting is becoming a combination of traditional Chinese and Western ideologies and practices [48]. It is time to reevaluate parenting practices in Chinese culture. Overall, our findings indicate that harsh parenting is not an optimal parenting practice in current Chinese society, and adolescent suicide ideation can be considered as an indication of ineffective parenting.

The result also revealed that adolescent self-esteem accounts for a significant proportion of the associations between harsh parenting and adolescent suicide ideation. The more harsh parenting the adolescents perceived, the more impaired are their self-esteem, which increase the risk of suicide ideation. In parallel with the integrated motivational-volitional model of suicidal behavior [51], stressful life experiences and consequent low self-esteem operate as vulnerability factors in the pre-motivational phase and predict suicide ideation during the motivational phase. Similarly, parental acceptance-rejection theory holds that individuals who perceived parental rejection are likely to define themselves as unworthy of love and develop a sense of overall negative self-evaluation [52]. They are also more disposed to experience behavior problems, depressive symptoms, and psychological disorders than accepted ones [53].

Thus, teenagers with low self-esteem may prone to consider suicide as an alternative to solve the distressing cognition and emotion connected to harsh parenting and negative self-concept, once again underscoring self-esteem as a protective factor.

The results revealed that school social support moderated the relationship between harsh parenting and self-esteem. Specifically, at the low level of harsh parenting, adolescents with high school social support had higher self-esteem than those with poor support, but it is worth noting that this advantage weakened as the level of harsh parenting grew. The results further support Luthar et al.’s idea of protective-reactive influence [54], which states that a particular protective factor only partially buffers the adverse effect of risk on youth maladjustment. One interpretation, based on the existential work of Vanderbilt-Adriance and Shaw (2008), is that despite the rising yearning for independence and reaching out into the wider social environment during adolescence, families remain primacy to youth and have significant effects on them [55]. Additionally, harsh parenting may not present in isolation but is bound up with other family risk factors. Considering only one category of risk is likely to underestimate the full impact of risk experienced by children and adolescents. Besides the cumulative character, the continuity nature of family environment risk also magnifies its destructive impact. The findings on protective-reactive effects indicate that in addition to acknowledging the limited protective role of school social support, we should also realize that decreasing the level of risk is particularly important for adolescent suicide prevention and intervention.

Another finding of this study is that the effect of self-esteem on reducing suicide ideation was stronger among adolescents with low versus high school social support. According to the threat-to-self-esteem model of support receipt [56], helping has both supportive and self-threatening components. The self-threatening components threaten the individual’s evaluation of their value and competence, resulting in more depressed moods and psychological distress. Individuals with high self-esteem are especially sensitive to the threatening aspects of help from others [57]. Social support may therefore become a source of pressure to hinder its potential efficiency as an adjustment resource. Results indicate that self-esteem and school social support are salient protective factors among adolescents that ameliorate the risk of suicide, nevertheless, it is worth recognizing the self-threatening aspect of social support.

Results obtained from this study should be interpreted within the context of several limitations. First, the findings are correlational due to the nature of the cross-sectional study. To examine the causality and bi-directionality among harsh parenting, self-esteem and suicide ideation in adolescents, additional longitudinal study is required. Second, the data were based on self-report, future research could benefit from using interview or observational data or collecting data from multiple sources to reduce the common method variance bias. Third, scales of harsh parenting in the current study measured the frequency but did not fully capture the intensity and duration of harsh parenting suffered by adolescents. Future research could choose a more comprehensive tool. What’s more, verbally-abusive and physically-abusive parenting have different associations with adolescent developmental outcomes. Further studies could explore their unique contribution to adolescent suicide ideation. Fourth, self-esteem can be broadly classified into explicit and implicit [28]. The current study only examined the link between harsh parenting and global self-esteem through explicit self-report methods, whether harsh parenting influences implicit self-esteem is unknown. Finally, the participants were sampled from towns and small cities in Hunan, China. More effort is needed to clarify whether our findings could be generalized to other areas or other nations.

Despite these shortcomings, this study not only provides insights into the potential ways in which harsh parenting be related to adolescent suicide ideation but also presents meaningful inspirations for developing adolescent suicide prevention and intervention strategies. The training designed to strengthen both self-esteem and school social support is appropriate. Given the evidence regarding the negative effects of harsh parenting and the limited buffering effect of school social support, it would be more effective to focus on prevention than on intervention to reduce the connection between harsh parenting and adolescent suicide ideation. Taking a family-focused perspective when developing prevention program is vital. Parents who employ harsh parenting should be provided with the necessary support to shift from harsh parenting practices to other nonviolent forms.

## Conclusions

The current study explores the mechanism underlying the relationship between harsh parenting and suicide ideation among Chinese adolescents. The results demonstrated that harsh parenting was a risk factor for adolescent suicide ideation and this association could be explicated partially by impaired self-esteem. School social support moderated the relationship between harsh parenting and self-esteem as well as the relationship between self-esteem and suicide ideation, but it is worth noting that this advantage weakened as the level of harsh parenting and self-esteem grew. The findings underscore the roles of harsh parenting and self-esteem to understand the shape of suicide ideation.

## Data Availability

The datasets used and/or analyzed during the current study are available from the corresponding author on reasonable request.
